# Impact of Sucrose Consumption on the Metabolic, Immune, and Redox Profile of Mice with Gestational Diabetes Mellitus

**DOI:** 10.3390/life15070989

**Published:** 2025-06-20

**Authors:** Cristian Ángel Rosales-Gómez, Beatriz Elina Martínez-Carrillo, Ana Laura Guadarrama-López, Aldo Arturo Reséndiz-Albor, Ivonne Maciel Arciniega-Martínez, Efrén Aguilar-Rodríguez

**Affiliations:** 1 Laboratorio de Investigación en Nutrición, Facultad de Medicina, Universidad Autónoma del Estado de México, Paseo Tollocan Esquina Jesús Carranza s/n, Colonia Moderna de la Cruz, Toluca C.P. 50180, Mexico; criztian13@yahoo.com.mx (C.Á.R.-G.); efren.aguilar.rod@gmail.com (E.A.-R.); 2 Clínica Multidisciplinaria de Salud, Universidad Autónoma del Estado de México, Jesús Carranza Esquina Venustiano Carranza No.205, Colonia Universidad, Toluca C.P. 50130, Mexico; alguadarramal@uaemex.mx; 3 Laboratorio de Inmunología de Mucosas, Sección de Estudios de Posgrado e Investigación, Escuela Superior de Medicina, Instituto Politécnico Nacional, Plan de San Luis y Díaz Mirón s/n, Colonia Casco de Santo Tomás, Alcaldía Miguel Hidalgo, Ciudad de México C.P. 11340, Mexico; alrealdo@yahoo.com.mx; 4 Laboratorio de Inmunonutrición, Sección de Estudios de Posgrado e Investigación, Escuela Superior de Medicina, Instituto Politécnico Nacional, Plan de San Luis y Díaz Mirón s/n, Colonia Casco de Santo Tomás, Alcaldía Miguel Hidalgo, Ciudad de México C.P. 11340, Mexico; ivonne.arciniega.77@gmail.com

**Keywords:** gestational diabetes mellitus, sucrose, immune response, redox mechanisms

## Abstract

Carbohydrate consumption during pregnancy represents an important source of energy; its consumption, however, can cause gestational diabetes mellitus (GDM), body weight gain, inflammation, increased glucose transport to the fetus, adiposity, and a risk of macrosomia. The objective was to research the impact of sucrose consumption during pregnancy on the metabolic, immune, and redox profile in female mice with GDM. A total of 24 female CD1 mice were used, divided into two groups: Control and GDM. Each group was subdivided into two subgroups: (a) Without sucrose and (b) With sucrose. The females were mated, and, once pregnancy was confirmed, GDM was induced by administering 230 mg/kg of streptozotocin subcutaneously. GDM was confirmed by glucose ≥ 200 mg/dL and the presence of polyphagia, polydipsia, and change in body weight. Metabolic, immune, and redox profile parameters were determined. Sucrose consumption groups increase HOMA-IR and the secretion of insulin, adiponectin, and leptin; it also increased the secretion of proinflammatory cytokines and the production of IgA and IgG antibodies, decreased the activity of the Glutathione Reductase enzyme, and increased the production of TBARS and AGE. High sucrose consumption increases the inflammatory response mediated mainly by CD8^+^ lymphocytes and the production of proinflammatory cytokines; it can trigger a compensatory humoral response and alter redox mechanisms, causing a state of Oxidant Stress.

## 1. Introduction

Diabetes mellitus (DM) is the most prevalent non-communicable metabolic disease, impacting more than 425 million people globally and causing 4.8 million deaths annually [1]. Approximately 28 million women of reproductive age have Type 2 DM (T2DM), predominantly in low- and middle-income countries [2,3,4]. The prevalence of DM during pregnancy, encompassing Type 1, Type 2, and gestational diabetes, ranges from 5% to 20%, with gestational DM (GDM) contributing 1% to 14% [3,5]. GDM can occur during pregnancy in women, regardless of personal or family medical history [6]. It leads to insulin resistance and results in sustained hyperglycemia, lipolysis, and hyperketonemia [7]. Factors influencing insulin resistance include elevated free fatty acids due to lipolysis and insufficient coupling between insulin receptor (IR) activation and GLUT4 receptor translocation to the cell surface [8].

A high consumption of simple carbohydrates (SCHs) or sugars during pregnancy poses a significant risk factor for expectant mothers [9]. Carbohydrates are a crucial energy source for both the mother and the developing fetus [10]. It is essential to consider both the quality and quantity of carbohydrate intake, particularly during gestational diabetes mellitus (GDM), as consuming simple sugars can rapidly elevate plasma glucose concentrations [11]. Evidence from animal models indicates that sucrose consumption in mothers with GDM negatively affects fetal development. It enhances glucose transport to the fetus, increases offspring adiposity, and raises the risk of macrosomia [12]. Additionally, GDM activates the Th1 and Th2 immune responses, leading to a pro-inflammatory reaction that contributes to a low-grade systemic inflammatory state [13,14,15]. During GDM, there is an increased secretion of pro-inflammatory Th1 cytokines, such as Interferon-γ (IFN-γ), Interleukin 2 (IL-2), and Tumor Necrosis Factor-α (TNF-α), which diminishes the Th2 response, particularly at implantation and childbirth [16]. Pro-inflammatory cytokines have been shown to disrupt insulin signaling and inhibit pancreatic beta-cell insulin release [15]. This disruption causes insulin resistance by decreasing the tyrosine kinase activity of the IR and increasing the serine phosphorylation of Insulin Receptor Substrate 1 (IRS-1) [17].

Conversely, the hyperglycemic and hyperlipidemic environment surrounding the embryo reduces antioxidant capacity and generates oxidizing substances, such as reactive oxygen species (ROS), which damage the structure of bioactive molecules, altering their functionality [18]. In patients with GDM, oxidative stress (OxS) affects the mother, placental function, and fetal well-being [19]. These complications are associated with the formation of advanced glycation end products (AGEs) [20]. ROS inhibits glucose uptake by phosphorylating ISR-1, which prevents the expression of GLUT4 receptors in the cell membrane, thereby maintaining a hyperglycemic state [21]. The objective of this study was to investigate the impact of sucrose consumption on the metabolic, immune, and redox profiles of mice with GDM.

## 2. Materials and Methods

### 2.1. Study Design

The experimental model included 24 CD1 female mice (n = 6), each 10 weeks old and pathogen-free. These mice were housed and handled in the Bioterium of the Faculty of Medicine at Universidad Autónoma del Estado de México (UAEMéx), following the guidelines of the Official Mexican Standard 062 [NOM-062-ZOO-199 Technical specifications for the production, care, and use of laboratory animals] [22] and adhering to the ARRIVE (Animal Research: Reporting of In Vivo Experiments) guidelines [23]. The protocol received approval from the Faculty’s Research Ethics Committee (CONBIOETICA-15-CEI-002-20210531). The mice were fed a standard diet (Rodent Laboratory Chow 5001 from Purina, providing 3.02 Kcal/g, Avon, CT, USA), with water supplied ad libitum. Two mice were housed per cage under controlled conditions, with temperatures maintained at 19–21 °C and 12 h light/dark cycles.

After gestation (between days 23 and 26 of the experiment), after the females had given birth to the pups, they were euthanized through an overdose of inhaled anesthetic in an ether chamber within an extraction hood, as specified in NOM-062-ZOO-199 section 9.4.1.2.1. This procedure was conducted within the first 6 h following the birth of the pups. After euthanasia, a whole-blood sample was collected intracardially using a heparinized syringe, yielding a total volume of 2 mL per female. From the whole blood, 1 mL was used to isolate peripheral lymphocytes [24] for the identification of cellular phenotypes and intracellular cytokines, employing the method previously described by Gutiérrez-Pliego et al. [25], with some modifications. The remaining samples were centrifuged at 2500 rpm for 10 min to separate the blood into two phases. The plasma was then collected with a Pasteur pipette and transferred to microcentrifuge tubes for subsequent processing to determine hormonal, metabolic, and humoral parameters, as well as redox capacity [24].

### 2.2. Study Groups

Ten-week-old female subjects were randomly selected and divided into four groups, each consisting of six individuals (n = 6): (1) a control group without sucrose supplementation [CL (−) sucrose], (2) a control group with sucrose supplementation [CL (+) sucrose], (3) a GDM group without sucrose supplementation [GDM (−) sucrose], and (4) a GDM group with sucrose supplementation [GDM (+) sucrose].

### 2.3. Gestational Diabetes Mellitus Induction

**Figure 1:**
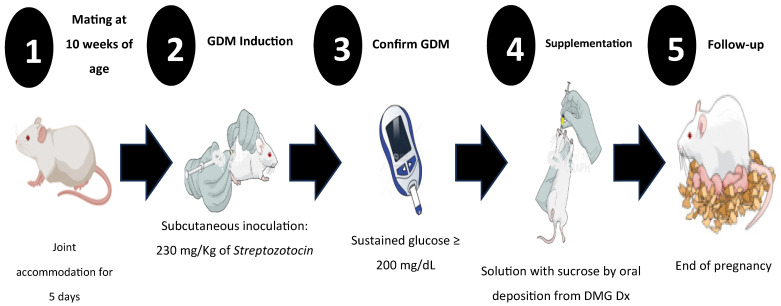
Experimental design for induction and follow-up of gestational diabetes mellitus (GDM), shown from the beginning to the end of pregnancy. Once GDM was induced, glycemia, weight, food intake, and daily water consumption were monitored to establish clinical signs of GDM and corroborate the diagnosis.

Before generating the GDM, the females were mated, with one male placed for every two females. On the fifth day of mating, once pregnancy was confirmed through the presence of a vaginal plug, DM was induced at 8 a.m. (Figure 1). To induce DM, ChemCruz^®^ (Cat. No. 18883-66-4, Dallas, TX, USA) Streptozotocin (U-9889) was administered subcutaneously in 0.5 M Citrate Buffer solution with a pH of 4.5 (VWR, Cat. No. J60024-AP, Ward Hill, MA, USA). The previously standardized dose was 230 mg/kg of weight [26]. DM diagnosis in all female mice was confirmed using criteria established by the American Diabetes Association (ADA) 2018: sustained glucose concentration ≥ 200 mg/dL, polyphagia, polydipsia, and changes in body weight [26].

### 2.4. Sucrose Supplementation

For groups receiving sucrose supplementation, a solution was prepared using ultrapure water and sucrose at a concentration of 41.66 mg/mL, following the guidelines of the Official Mexican Standard NOM-218-SSA1-2011 for non-alcoholic flavored beverages and related products [27]. Each female received a daily oral dose of 500 µL of this solution administered to each female by oral deposition daily at 8:00 am, from the confirmation of gestational diabetes until the end of pregnancy.

### 2.5. Calculation and Determination of Metabolic Parameters

Weight and body mass index (BMI) Quantification: Weight was measured weekly using an Ohaus™ Triple Beam 700/800 Series mouse weighing scale (Cat. No. 2,729,439, Morris, NJ, USA) from the start of sucrose supplementation through to the end of gestation. The BMI was calculated using the following formula:

$$BMI=\frac{Weight\left(g\right)}{{Length\;(cm)}^{2}}$$

Glycemia Quantification: Each week, blood glucose levels were measured using the One Touch^®^ Brand Select Plus Flex Glucometer (Cat. No. AW06984804A, Zug, Switzerland). Measurements were obtained through the capillary puncture of the tail vein, starting at the onset of sucrose supplementation and continuing until the end of pregnancy.

Hormone and HOMA-IR Index: Adiponectin, leptin, and insulin concentrations were quantified using Enzyme-Linked Immunosorbent Assay (ELISA) with commercial kits. For leptin and insulin, ENZO brand kits were utilized (leptin: Cat. No. ADI-900-119A; insulin: Cat. No. EZN-KIT141-000, Farmingdale, NY, USA). For adiponectin, a kit from Invitrogen was employed (Cat. No. KMP0041, Vienna, Austria), following the suppliers’ instructions.

Using the data collected on insulin and glycemia, we calculated the HOMA Index (Homeostasis Model Assessment—Insulin Resistance Index) to evaluate the degree of insulin resistance. The calculation was performed using the following formula [28]:

$$HOMA=\frac{\left[\left(Glycemia\;(mg/dL)\right)\;/\;18.2)\;*\;Insulin\;(mU/dL)\right]}{22.517}$$

### 2.6. Immunity Components Quantification

Cell Population and Cytokine Secretion Analysis: To quantify immune cell populations and cytokine secretion in peripheral blood, we employed monoclonal antibodies directly conjugated with fluorochromes. The antibodies were sourced from Becton Dickinson, including CD3 (PE, Cat. No. 553063, Franklin Lake, NJ, USA) and CD8 (APC, Cat. No. 553035, Franklin Lake, NJ, USA). Additionally, antibodies from BioLegend were used: CD4 (PerCP, Cat. No. 100538, San Diego, CA, USA), IL-1β (FITC, Cat. No. 506907, San Diego, CA, USA), IL-6 (APC, Cat. No. 504504, San Diego, CA, USA), IFG-γ (FITC, Cat. No. 505806, San Diego, CA, USA), and TNF-α (PE, Cat. No. 506306, San Diego, CA, USA). The analysis was conducted using a Becton Dickinson FACS Aria Flow Cytometer, with 100,000 events counted per tube. The data were subsequently processed using Summit V6 software.

Immunoglobulin concentration in serum: Immunoglobulin concentration in serum was determined by quantifying specific antibodies through the ELISA method, as described by Carrizales et al. [29]. The antibodies used were Goat Anti-Mouse IgA HRP from US Biological (Cat. No. I1890-21, Salem, MA, USA) and Peroxidase-Conjugated Goat IgG Fraction to Mouse (Cat. No. 55553, Solon, OH, USA).

### 2.7. Redox Capacity Quantification

For the reduction systems, we quantified total antioxidant capacity and reduced glutathione using commercial kits from QuantiChrom™ (Cat. No. DTAC-100 and Cat. No. DIGT-250, respectively, Hayward, CA, USA). Additionally, EnzyChrom™ catalase and superoxide dismutase kits (Cat. No. ECAT-100 and Cat. No. ESOD-100, Hayward, CA, USA) were used, following the manufacturer’s instructions.

For oxidation systems, reactive species of thiobarbituric acid were quantified using a commercial kit from QuantiChrom™ (Cat. No. DTBA-100, Hayward, CA, USA). To quantify advanced glycation end products, the Carbonylated Proteins method was employed [30].

### 2.8. Statistical Analysis

The data were presented using measures of central tendency. We assessed data normality with the Shapiro–Wilk test. To compare differences between groups, we analyzed homogeneous results using a one-way analysis of variance (ANOVA) with Tukey’s post hoc test for independent samples. For non-homogeneous variables, we employed the Kruskal–Wallis statistic with the Games–Howell post hoc test to identify intergroup differences. The analysis was conducted using the Statistical Package for the Social Sciences software (SPSS, version 19.0; SPSS Inc., Chicago, IL, USA). A *p* value of less than 0.05 was considered statistically significant.

## 3. Results

### 3.1. Morphometric Values and Glycemia

At the beginning of the 10th week of the females’ development, upon confirming the diagnosis of GDM and initiating sucrose supplementation, weight and BMI measurements were recorded. The results were as follows: CL (−) sucrose group showed a weight of 30.72 ± 1.91 g and a BMI of 0.307 ± 0.02 g/cm^2^; CL (+) sucrose group had a weight of 28.3 ± 0.11 g and a BMI of 0.272 ± 0.01 g/cm^2^; GDM (−) sucrose group recorded a weight of 24.28 ± 2.55 g and a BMI of 0.221 ± 0.03 g/cm^2^; GDM (+) sucrose group had a weight of 28.5 ± 1.34 g and a BMI of 0.265 ± 0.01 g/cm^2^. By the end of gestation, the GDM (−) sucrose group maintained the lowest weight and BMI (F = 22.539, *p* < 0.001 for weight, and F = 24.980, *p* < 0.001 for BMI), followed by the GDM (+) sucrose and CL (+) sucrose groups, in comparison to the CL (−) sucrose group (F = 24.280, *p* < 0.001) (Table 1).

The median blood glucose concentration across all groups before the induction of GDM was 140 mg/dL. After sucrose supplementation commenced (at 10 weeks of age) and pregnancy was confirmed, significant differences emerged between the groups (F = 85.971, *p* < 0.001). The median glycemia levels were as follows: CL (−) sucrose, 155 mg/dL; CL (+) sucrose, 146.5 mg/dL; GDM (−) sucrose, 533 mg/dL; and GDM (+) sucrose, 600 mg/dL. By the end of pregnancy, significant differences persisted between the groups (F = 64.917, *p* < 0.001). The GDM (+) sucrose group exhibited the highest glycemia values, followed by the GDM (−) sucrose group. The CL (+) sucrose group had significantly elevated glycemia (F = 64.917, *p* < 0.001) compared to the CL (−) sucrose group, though these levels were not as high as those in the GDM groups (Table 1).

### 3.2. Metabolic Parameters

Insulin, Adiponectin, Leptin, and HOMA-IR index: The concentrations of insulin (F = 0.027, *p* = 0.994) and adiponectin (F = 1.827, *p* = 0.175) did not differ significantly at either the beginning or end of supplementation and gestation (Table 2). Initial HOMA-IR values also showed no differences (F = 2.877, *p* = 0.062); however, by the end of gestation, both the GDM (−) sucrose and GDM (+) sucrose groups exhibited significant increases (post hoc Tukey, F = 65.764, *p* < 0.001) compared to the CL (−) sucrose and CL (+) sucrose groups (Table 2). In contrast, leptin levels showed differences between groups both at the beginning (F = 14.065, *p* < 0.001) and the end of pregnancy and supplementation (F = 6.485, *p* < 0.003). Initially, the CL (+) sucrose group had the highest leptin concentrations, followed by the GDM (+) sucrose group. At the experiment’s conclusion, the CL (+) sucrose group maintained high leptin levels, while the GDM (−) sucrose group exhibited the lowest values of this hormone (Table 2).

### 3.3. Immune Parameters

Percentage of T Lymphocytes: The T lymphocyte populations were measured to determine changes. An increase in TCD3^+^ lymphocytes was observed in groups supplemented with sucrose, both CL (+) sucrose and GDM (+) sucrose (F = 6.088, *p* < 0.004), compared to the CL (−) sucrose group, with no significant changes noted in the GDM (−) sucrose group. For the TCD3^+^/CD4^+^ lymphocyte population, there was a notable decrease (F = 20.813, *p* < 0.001) in both CL (+) sucrose and GDM (+) sucrose groups when compared to the CL (−) sucrose and GDM (−) sucrose groups (Table 3). Conversely, TCD3^+^CD8^+^ lymphocytes increased significantly (F = 39.615, *p* < 0.001) only in the CL (+) sucrose group, as opposed to all CL (−) sucrose groups and both GDM groups, regardless of sucrose consumption (Table 3).

Interleukin 1β (IL-1β) expression increased exponentially in the CL (+) sucrose, GDM (−) sucrose, and GDM (+) sucrose groups compared to the CL (−) sucrose group (F = 1294.801, *p* < 0.001) (Table 3). Interleukin 6 (IL-6) levels were significantly higher in the sucrose-consuming groups, CL (+) sucrose and GDM (+) sucrose, than in the CL (−) sucrose group (F = 708.587, *p* < 0.001) (Table 3). Additionally, the cytokines INF-γ and TNF-α showed substantial elevation in the GDM (+) sucrose group, with F-values of 837.181 (*p* < 0.001) and 1741.175 (*p* < 0.001), respectively (Table 3).

Serum immunoglobulins: Serum immunoglobulins A (IgA) and G (IgG) were quantified, with absorbance measurements conducted at a wavelength of 492 nm and a protein concentration of 25 µg/mL per sample. For IgA, the highest concentration was observed in the GDM (−) sucrose group (F = 6.950, *p* < 0.002), followed by the GDM (+) sucrose group. These levels were compared with those in the CL (+) sucrose and CL (−) sucrose groups. Conversely, IgG levels increased significantly in the GDM (−) sucrose group (F = 4.288, *p* < 0.017), then in the CL (+) sucrose and GDM (+) sucrose groups, when compared to the CL (−) sucrose group (Table 3).

### 3.4. Redox Activity

Antioxidant Parameters: The antioxidant systems were altered, with the exception of catalase, which did not differ significantly between the groups (X^2^ = 5.507, *p* = 0.138). Total Antioxidant Capacity was highest in the GDM (+) sucrose group, followed by the CL (+) sucrose and CL (−) sucrose groups, compared to the CL (−) sucrose group (X^2^ = 9.385, *p* = 0.025). The superoxide dismutase enzyme levels were elevated in both GDM (+) sucrose and GDM (−) sucrose groups but decreased in the CL (+) sucrose group (X^2^ = 4.717, *p* = 0.194). Lastly, the activity of Glutathione Reductase decreased across all groups (X^2^ = 2.384, *p* = 0.497) (Table 4).

Oxidant Parameters: Contrary to antioxidant capacity, the oxidant systems changed with sucrose supplementation. Advanced glycation end products (AGE) increased in the GDM (+) sucrose and CL (+) sucrose groups, while they decreased in the CL (−) sucrose group (X^2^ = 20.534, *p* < 0.001). Conversely, the production of Thiobarbituric Acid-Reactive Species (TBARS) rose in the GDM (−) sucrose group but decreased in both the CL (+) sucrose and CL (−) sucrose groups (X^2^ = 12.415, *p* = 0.006) (Table 4).

## 4. Discussion

Pregnancy is a transitional state marked by adaptive and compensatory changes that cater to the specific needs of each trimester [31]. During this process, there is an increase in adipose tissue, driven by the release of placental hormones, which reduces insulin sensitivity by up to approximately 60% by the second trimester [32]. Glucose metabolism also changes with gestation to meet the fetus’s growing energy demands [33]. Initially, fasting glucose levels may decrease due to increased maternal plasma volume; however, this trend reverses as maternal adipose tissue stores increase, heightening the risk of GDM [34]. This metabolic condition leads to an elevated risk of comorbidities for both mother and fetus [35]. For the mother, there is an increased risk of hypertension, preeclampsia, cesarean delivery, and premature birth [36]. For the fetus, the risks include dystocia, respiratory distress syndrome, jaundice, and neonatal hyperglycemia [37].

### 4.1. Changes in Weight and BMI Related to GDM

Weight gain during pregnancy is influenced by both the trimester of gestation and the mother’s dietary intake, with carbohydrates being the primary energy source for pregnant women [38]. Simple carbohydrates, in particular, are associated with rapid increases in blood glucose levels after consumption, leading to significant postprandial hyperglycemia [39].

There is no definitive data on the relationship between simple carbohydrate intake and weight gain during pregnancy. Conversely, it has been reported that patients with gestational diabetes tend to gain less weight compared to those without diabetes, which may correlate with the birth weight of the infant [40]. This study’s findings align with this observation; both the GDM group and the sucrose-supplemented control group experienced less weight gain than the control group without supplementation. Moreover, the BMI was lower in sucrose-supplemented groups subjected to high glucose loads, which induced hyperglycemic states (Table 1).

### 4.2. Insulin Resistance and GDM

Insulin resistance is influenced by various factors such as age, race, BMI, and, for women, the number of births [41]. It has been reported that, during pregnancy, a weight gain of 5 kg can increase HOMA-IR, indicating that higher BMI levels correlate with greater insulin resistance in pregnant women [42,43].

In GDM, insulin resistance is closely linked to deficient insulin secretion by both the pancreatic beta cells and the adipose tissue of pregnant women [44]. Studies comparing postprandial insulin secretion in patients with GDM, normal weight, and obesity to those without GDM and healthy pregnancies indicate an increase in insulin levels and resistance in patients with GDM and obesity. Conversely, patients with normal weight or who are thin, even with GDM, demonstrate lower insulin concentrations, sometimes even lower than those with healthy pregnancies [45,46]. The findings from this study align with these reports, showing greater insulin resistance among groups with GDM, regardless of weight or BMI. Notably, sucrose supplementation did not significantly impact insulin secretion; the non-GDM group supplemented with sucrose exhibited the lowest insulin concentration by the study’s end. These results may support the adaptive changes during pregnancy in response to insulin resistance, where the pancreas enlarges due to alterations in the mass and function of pancreatic beta cells [47]. This adaptation helps to manage insulin resistance and maintain normal plasma glucose levels, highlighting a marked neogenesis of beta cells in the islets of Langerhans [48].

Inoue et al. [49] compared insulin secretion and insulin resistance between thin women with GDM and those without it. Their findings support the theory of adaptive changes in pancreatic beta cells. The study revealed that women with GDM had lower insulin sensitivity, while the group without GDM showed no significant changes [49].

### 4.3. Metabolic Changes During GDM

Insulin resistance and sensitivity are not the sole metabolic changes associated with GDM. Pregnancy induces metabolic stress characterized by alterations in adipose tissue, which produces adiponectin and leptin. These hormones are crucial for managing hunger and satiety, thereby regulating energy reserve needs [50]. Adiponectin is involved in energy regulation and plays a significant role in modulating the immune response. Some studies indicate that macrophages can express adiponectin, influencing metabolism in distant tissues and the inflammatory response [51,52]. In clinical studies involving animal models of GDM, an inverse correlation with adiponectin levels has been observed; reductions in this hormone are accompanied by increases in TNF-α and Interleukin-6 [53]. Furthermore, a lower concentration of adiponectin can exacerbate insulin resistance [54,55].

Leptin is released by healthy adipose tissue to regulate satiety at the hypothalamic level [56]. During pregnancy, the placenta assumes a prominent role in leptin production, facilitating amino acid transport to the fetus [57]. Early in pregnancy, leptin levels increase to regulate gonadotropin-releasing hormone and support embryo implantation, as well as fetal growth, development, and organ formation [58]. Studies indicate that leptin concentrations are elevated in GDM but decrease as pregnancy progresses; notably, patients with GDM exhibit higher leptin levels than those without GDM [59]. Our study’s metabolic analysis revealed decreased levels of both hormones in groups with GDM, particularly leptin, showing a significant reduction in secretion. This finding aligns with the observed higher HOMA-IR index in GDM groups compared to control groups, even among individuals with a lower BMI, underscoring the link between leptin dynamics and insulin resistance.

### 4.4. Immune Response in GDM

From the early stages of pregnancy until delivery, the immune system undergoes modifications to maintain a balance among its components, preventing the rejection of the offspring [60]. Hyperglycemia, whether or not accompanied by pregnancy, is known to cause immune system dysfunction. It adversely affects neutrophil chemotaxis, amplifies the inflammatory response of macrophages, and reduces phagocytic function. This dysfunction is exacerbated when pregnancy is accompanied by GDM, thereby increasing the risk of infections and comorbidities in affected individuals [61,62].

Evidence indicates the infiltration of CD8^+^ and CD4^+^ T cells in visceral adipose tissue, prompting inflammation and activating M1 macrophages. This activation leads to the secretion of pro-inflammatory cytokines, such as TNF-α and IL-6, which increase insulin resistance in GDM [63]. Other studies report an elevated expression of the Th1 transcription factor and heightened production of cytokines IL-2 and INF-γ in women with obesity and GDM, suggesting that the inflammatory state in this condition results from an imbalance in the Th1/Th2/Treg ratio [64,65]. These findings confirm that the hyperglycemic state during GDM can exacerbate the pro-inflammatory response, particularly with the ingestion of simple sugars like sucrose. In our study, we observed that the percentage of CD3^+^/CD8^+^ lymphocytes and the production of pro-inflammatory cytokines were higher in groups with sucrose consumption—specifically, CL (+) sucrose and GDM (+) sucrose. This increase may imply an effect of sucrose on the immune system, though it may not relate directly to GDM.

### 4.5. Changes in Humoral Response During GDM

B lymphocytes are responsible for antibody production, providing specific protection against antigens and contributing to autoimmune inflammatory diseases [66]. Research indicates that obesity and hyperglycemia directly influence antibody production and promote insulin resistance [67]. In obese mice, immunoglobulin G (IgG) production exceeds that in lean mice, suggesting involvement in regulating insulin resistance through macrophages infiltrated in visceral adipose tissue [68,69]. Most immune cells primarily use oxidative phosphorylation in mitochondria to generate energy efficiently. When activated by an antigenic challenge, they shift to aerobic glycolysis for a faster response [70,71]. This increased glycolytic flux directly activates major signaling pathways, such as the NF-κB (Nuclear Factor kappa B) pathway. Intermediate glucose metabolites can activate protein complexes like Complex IkB kinase (IKK), which releases NF-κB, thereby activating the transcription of genes for pro-inflammatory cytokines, including Tumor Necrosis Factor-alpha (TNF-α), Interleukin-6 (IL-6), and Interleukin-1 beta (IL-1β) [72,73].

In a separate study, researchers analyzed the antibody parameters of 124 pregnancies with GDM and 168 pregnancies without diabetes using flow cytometry. The findings revealed a significantly higher percentage of B lymphocytes and increased IgA production in pregnancies with GDM compared to those without GDM [74]. Elevated IgA levels have also been linked to adipose tissue inflammation and glucose homeostasis, contributing to insulin resistance associated with obesity and overweight [75]. Although research has concentrated mainly on circulating B lymphocyte populations during GDM, it has paid limited attention to T lymphocyte populations. Notably, a marked increase in B lymphocytes is positively associated with insulin resistance and elevated IgA production [76].

The findings of this study align with previous reports on antibody production during gestational diabetes. However, it was observed that sucrose consumption, rather than hyperglycemia, in females with GDM and the sucrose-consuming CL (+) group was responsible for the increase in IgA and IgG antibody secretion. Notably, IgG levels increased significantly in both the CL (+) sucrose and DMG (+) sucrose groups. Despite these findings, limited information exists regarding changes in the humoral or cellular immune status in patients with GDM and their relationship with the consumption of simple carbohydrates both before and during pregnancy. Furthermore, the influence of these factors on diagnosis and complications is not well-understood.

### 4.6. Modifications of Redox Mechanisms During GDM

Hyperglycemic states can trigger various metabolic disorders related to oxidative stress [77]. These states disrupt the redox system balance by activating the enzyme NADPH oxidase, facilitating the advanced glycation of proteins and lipids, activating Protein Kinase C, and promoting the formation of glycosaminoglycans [78]. Oxidative stress significantly impacts the structural changes of proteins, lipids, and nucleic acids [79]. ROS can oxidize low-density lipoproteins (LDL) and damage endothelial cells through adhesion molecules like ICAM-1 and VCAM-1 [80]. This ROS production is further exacerbated by high sucrose intake. In animal models on sucrose-rich diets, ROS production increases due to accelerated oxidation in hepatocytes, while antioxidant processes in the blood decrease [81,82,83]. Another study found that sucrose-rich diets initially trigger an antioxidant response through the increased activity of superoxide dismutase (SOD), catalase, and glutathione peroxidase (GPx). However, chronic sucrose consumption over eight weeks resulted in reduced antioxidant response, with a decreased activity of SOD and catalase and a milder effect on GPx [84].

A diet rich in sucrose consumed over a 2-week period increases TBARS production, indicating heightened lipid peroxidation, while exhibiting decreasing SOD levels and showing no changes in GPx activity. These findings suggest that high sucrose intake quickly disrupts redox balance, leading to oxidative stress [85]. In this study, hyperglycemia and sucrose consumption increased total antioxidant capacity and SOD activity, with minimal changes in catalase activity and a reduction in Glutathione Reductase activity. These data align with oxidation mechanisms linked to prolonged sucrose consumption, which elevates TBARS and AGE production, regardless of the presence of GDM. Notably, AGE generation was lower in the diabetes group without supplementation compared to other study groups.

## 5. Conclusions

GDM is closely linked to a low-grade inflammatory process. Pregnancy itself involves adaptive changes; surpassing these changes can trigger alterations in metabolic, immunological, and redox states. High sucrose consumption significantly contributes to the exacerbation and persistence of these adverse effects. When combined with the presence of GDM, the harm to both the pregnant patient and the offspring increases considerably. In this context, alterations in the secretion of adiponectin and leptin have been confirmed, along with an enhanced inflammatory response primarily mediated by TCD8^+^ lymphocytes, leading to the production of pro-inflammatory cytokines.

Furthermore, a compensatory humoral response is evident through the production of IgA and IgG antibodies. This response alters redox mechanisms, leading to oxidative stress and the consequent production of advanced glycation end products (AGEs) and Thiobarbituric Acid-Reactive Species (TBARS). This condition collectively increases comorbidities during pregnancy and directly impacts the child’s growth and development. However, it is necessary to determine whether these changes persist post-delivery in both the mother and the child, and if the humoral response generates specific antibodies against hyperglycemia.

## Figures and Tables

**Table 1:** Morphometric and glycemic data in CD1 females with and without sucrose supplementation at the end of pregnancy.

	CL (−) Sucrose	CL (+) Sucrose	DMG (−) Sucrose	DMG (+) Sucrose	
	Mean ± SD (*n* = 6)	Mean ± SD (*n* = 6)	Mean ± SD (*n* = 6)	Mean ± SD (*n* = 6)	*p* * Value
Weight (g)	31.52 ± 2.67	27.65 ± 0.60	22.60 ± 1.68	25.38 ± 2.19	0.001 *
BMI (g/cm^2^)	0.315 ± 0.03	0.263 ± 0.01	0.220 ± 0.02	0.233 ± 0.02	0.001 *
	Median	Median	Median	Median	
Glycemia (mg/dL)	207	219	577	586	0.001 *

Glycemia (mg/dL) measured at the end of pregnancy (the end of gestation between day 23 and 26; 13-week-old female mice). For homogeneous data, one-way ANOVA was performed and, for glycemia (non-homogeneous data), the non-parametric Kruskal–Wallis test was performed to compare the differences between groups. In both cases, the differences were considered statistically significant with a *p* value < 0.05 *. Control without sucrose supplementation (CL (−) sucrose), CL with sucrose supplementation (CL (+) sucrose), gestational diabetes mellitus without sucrose supplementation (GDM (−) sucrose), gestational diabetes mellitus with sucrose supplementation (GDM (+) sucrose). The samples were obtained between days 23 and 26 of the female’s pregnancy, at the end of supplementation, and at the birth of the pups.

**Table 2:** Metabolic parameters of CD1 females with and without sucrose supplementation at the beginning and end of gestation.

	CL (−) Sucrose	CL (+) Sucrose	DMG (−) Sucrose	DMG (+) Sucrose	
	Mean ± SD (*n* = 6)	Mean ± SD (*n* = 6)	Mean ± SD (*n* = 6)	Mean ± SD (*n* = 6)	*p* Value *
* 10th day, beginning of supplementation					
Insulin (pg/mL)	0.743 ± 0.012	0.743 ± 0.012	0.743 ± 0.008	0.743 ± 0.008	0.994
HOMA-IR Index	0.239 ± 0.257	0.2557 ± 0.257	0.209 ± 0.031	0.226 ± 0.029	0.062
Adiponectin (pg/mL)	8.5178 ± 1.031	8.76 ± 0.883	9.29 ± 2.4	10.53 ± 0.751	0.102
Leptin (pg/mL)	0.058 ± 0.031	0.074 ± 0.009	0.028 ± 0.012	0.038 ± 0.009	0.001 *
^&^ 23rd day, end of gestation and supplementation					
Insulin (pg/mL)	0.438 ± 0.018	0.436 ± 0.014	0.443 ± 0.024	0.445 ± 0.005	0.792
HOMA-IR Index	0.394 ± 0.1564	0.397 ± 0.1050	1.026 ± 0.0718	1.027 ± 0.0886	0.001 *
Adiponectin (pg/mL)	9.574 ± 0.778	7.792 ± 2.154	6.622 ± 3.139	7.631 ± 2.162	0.175
Leptin (pg/mL)	0.077 ± 0.029	0.101 ± 0.051	0.027 ± 0.018	0.043 ± 0.018	0.003 *

The values represent the mean ± standard deviation (SD) of insulin, adiponectin, and leptin in and HOMA-IR (Homeostasis Model Assessment—Insulin Resistance Index). The one-way ANOVA statistic was performed to compare the differences between the groups. The differences were considered statistically significant with a *p* value < 0.05 *. Picograms per milliliter (pg/mL), Control without sucrose supplementation (CL (−) sucrose), CL with sucrose supplementation (CL (+) Ssucrose), gestational diabetes mellitus without sucrose supplementation (GDM (−) sucrose), gestational diabetes mellitus with sucrose supplementation (GDM (+) sucrose). The samples were obtained: * Day 10, Confirmation of GDM, start of sucrose supplementation; **^&^** The end of gestation between day 23 and 26; 13-week-old female mice, end of supplementation, and birth of the pups.

**Table 3:** Immune parameters of female CD1 mice at the end of pregnancy.

	CL (−) Sucrose	CL (+) Sucrose	DMG (−) Sucrose	DMG (+) Sucrose	
	Mean ± SD (*n* = 6)	Mean ± SD (*n* = 6)	Mean ± SD (*n* = 6)	Mean ± SD (*n* = 6)	*p* Value *
Cell Immunity					
CD3^+^ (%)	26.41 ± 0.85	27.59 ± 0.71	26.05 ± 0.42	27.46 ± 0.95	0.004 *
CD3^+^/CD4^+^ (%)	44.45 ± 0.77	42.80 ± 0.43	44.15 ± 0.91	41.78 ± 0.41	0.001 *
CD3^+^/CD8^+^ (%)	26.37 ± 0.79	30.40 ± 0.85	27.05 ± 0.30	26.92 ± 0.78	0.001 *
IL-1β (pg/mL)	0.46 ± 0.20	8.28 ± 0.35	10.63 ± 0.56	12.50 ± 0.20	0.001 *
IL-6 (pg/mL)	1.08 ± 0.16	10.89 ± 0.80	7.39 ± 0.11	10.72 ± 0.19	0.001 *
INF-γ (pg/mL)	0.20 ± 0.09	5.04 ± 0.46	6.36 ± 0.24	10.76 ± 0.51	0.001 *
TNF-α (pg/mL)	0.33 ± 0.14	4.31 ± 0.08	5.49 ± 0.18	8.32 ± 0.31	0.001 *
Humoral Immunity					
IgA (OD)	0.298 ± 0.037	0.537 ± 0.078	1.061 ± 0.627	0.939 ± 0.176	0.002 *
IgG (OD)	0.247 ± 0.093	0.421 ± 0.080	0.545 ± 0.249	0.422 ± 0.083	0.017 *

The values represent the mean ± standard deviation (SD) of the percentage of lymphocyte populations, concentration of cytokines and absorbances in Optical Densities (ODs) of immunoglobulins A and G. The one-way ANOVA statistic was performed to compare the differences between the groups. The differences were considered statistically significant with a *p* value < 0.05 *. Interleukin-1 βeta (IL-1β), Interleukin-6 (IL-6), Tumor Necrosis Factor-alpha (TNF-α), Interferon-gamma (INF-γ), picograms per milliliter (pg/mL), control without sucrose supplementation (CL (−) sucrose), CL with sucrose supplementation (CL (+) sucrose), gestational diabetes mellitus without sucrose supplementation (GDM (−) sucrose), gestational diabetes mellitus with sucrose supplementation (GDM (+) sucrose). The end of gestation between days 23 and 26; 13-week-old female mice, end of supplementation, and birth of the pups.

**Table 4:** Redox activity of female CD1 mice at the end of gestation.

	CL (−) Sucrose	CL (+) Sucrose	DMG (−) Sucrose	DMG (+) Sucrose	
	Median (*n* = 6)	Median (*n* = 6)	Median (*n* = 6)	Median (*n* = 6)	*p* * Value
Antioxidant Parameters					
TAC (µM)	0.133	0.167	0.159	0.179	0.025 *
Catalase (U/L)	0.182	0.168	0.193	0.172	0.138
SOD (U/mL)	1.274	0.925	1.823	2.047	0.019 *
GR (µM)	98.86	92.06	87.53	83	0.049 *
Oxidant Parameters					
TBARS (µM)	0.0218	0.0075	0.0625	0.1430	0.006 *
AGE (pM/dL)	1218	1791	725	3034	0.001 *

The values represent the median of antioxidant enzymes and oxidants of CD1 pregnant female mice. Total Antioxidant Capacity (TAC µM), catalase (U/L), superoxide dismutase (SOD U/mL), Glutathione Reductase (GR µM), U/mL), Thiobarbituric Acid-Reactive Species (TBARS µM), and products’ advanced glycation end products (AGEs pM/dL). The Kruskal–Wallis statistics were performed to compare the differences between the groups. The differences were considered statistically significant with a *p* value < 0.05 *. Control without sucrose supplementation (CL (−) sucrose), CL with sucrose supplementation (CL (+) sucrose), gestational diabetes mellitus without sucrose supplementation (GDM (−) sucrose), gestational diabetes mellitus with sucrose supplementation (GDM (+) sucrose). The samples were obtained on days at the end of gestation between days 23 and 26; 13-week-old female mice, end of supplementation, and birth of the pups.

## Data Availability

The data is available, if necessary; send a request via email to the corresponding author.

## Abbreviations

DM	Diabetes Mellitus
T1DM	Type 1 Diabetes Mellitus
T2DM	Type 2 Diabetes Mellitus
GDM	Gestational Diabetes Mellitus
IR	Insulin Receptor
ISR-1	Insulin Receptor Substrate 1
GLUT4	Glucose Transporter Protein Type 4
BMI	Body Mass Index
IL-1β	Interleukin-1 Beta
IL-6	Interleukin-6
INF-γ	Interferon Gamma
TNF-α	Tumor Necrosis Factor-Alpha
ROS	Reactive Oxygen Species
OxS	Oxidant Stress
AGEs	Advanced Glycation End Products
TBARS	Thiobarbituric Acid Reactive Species
ARRIVE	Animal Research: Reporting of In Vivo Experiments
CL (−)	Control Group without Sucrose Supplementation
CL (+)	Control Group with Sucrose Supplementation
GDM (−)	Gestational Diabetes Mellitus group without Sucrose Supplementation
GDM (+)	Gestational Diabetes Mellitus group with Sucrose Supplementation
ELISA	Enzyme-Linked Immunosorbent Assay
HOMA Index	Homeostasis Model Assessment—Insulin Resistance Index
TAC	Total Antioxidant Capacity
SOD	Superoxide Dismutase
IgA	Immunoglobulin A
IgG	Immunoglobulin G
